# Description of breed ancestry and genetic health traits in arctic sled dog breeds

**DOI:** 10.1186/s40575-021-00108-z

**Published:** 2021-09-20

**Authors:** Joseph A. Thorsrud, Heather J. Huson

**Affiliations:** Department of Animal Sciences, Cornell University College of Agriculture and Life Sciences, 201 Morrison Hall, 507 Tower Road, Ithaca, NY 14853 USA

**Keywords:** Dog Breeds, Genetic Health, Breed Ancestry, Alaskan Husky

## Abstract

**Background:**

This study describes the presence and frequency of health traits among three populations of dogs traditionally used for sledding and explores their ancestry and breed composition as provided by the commercially available Embark dog DNA test. The three populations include the purebred Siberian Husky and the admixed populations of Alaskan sled dogs and Polar Huskies. While the Siberian Husky represents a well-established breed with extensive historical and health data, the Alaskan sled dog is less studied but has been the subject of nutritional, physiological, and genetic studies related to ancestry and performance. In contrast, the Polar Husky is a relatively obscure and rare group of dogs used for arctic exploration with very little-known information. The three populations were compared using Embark results, providing new insight into the health traits circulating within the populations and the potential ancestral linkage of the health traits between the sledding populations. Embark results are based upon 228,588 single-nucleotide polymorphisms (SNPs) spanning the canine genome, characterized using a custom-designed Illumina beadchip array.

**Results:**

Specifically, breed composition was summarized for the two admixed populations with most of the dogs being predominantly categorized as Alaskan husky- type dog or “Supermutt”. Mitochondrial and Y chromosome haplogroups and haplotypes were found with Alaskan sled dogs carrying most of the haplogroups and types found in Siberian and Polar Huskies. Genomic principal component analysis reflected population structure corresponding to breed and substructure within the Alaskan sled dogs related to sprint or distance competition. Genetic markers associated with Alanine Aminotransferase activity, Alaskan Husky Encephalopathy, dilated cardiomyopathy, Collie eye anomaly, degenerative myelopathy, ichthyosis, and factor VII deficiency were identified in the populations of sledding breeds.

**Conclusion:**

These results provide a preliminary description of genetic characteristics found in sledding breeds, improving the understanding and care of working sled dogs.

**Supplementary Information:**

The online version contains supplementary material available at 10.1186/s40575-021-00108-z.

## Summary

When populations of dogs are bred for specific purposes such as sledding, it can lead to the accumulation of different traits unique to that group. This study examines the health traits, and genetic markers which differentiate between groups of Alaskan sled dogs, Siberian Huskies, and Polar Huskies which are all dog breeds selected for arctic sledding. The Alaskan sled dogs were found to have mutations related to Alanine Aminotransferase (ALT) activity, Alaskan Husky encephalopathy, Collie eye anomaly, degenerative myelopathy, dilated cardiomyopathy, factor VII deficiency, and ichthyosis. Siberian Huskies tested had mutations related to ALT activity, and Collie eye anomaly. Polar Huskies had the same mutations for ALT activity, Collie eye anomaly, and ichthyosis.

## Background

**Fig. 1:**
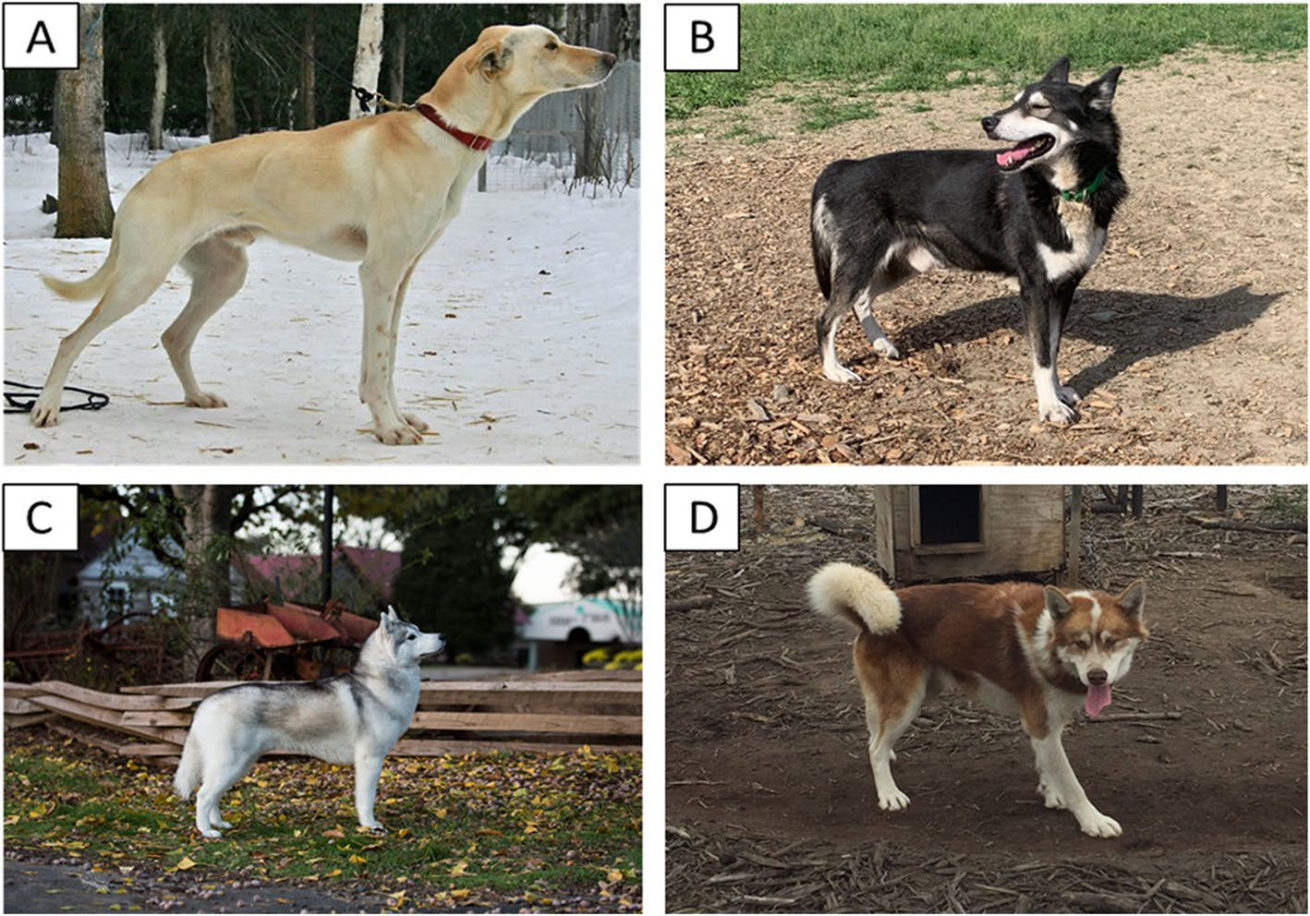
Photos of dogs representing the different sledding populations and subpopulations. **A** Alaskan sled dog bred for sprint racing. **B** Alaskan sled dog bred for distance racing. **C** Siberian Husky. **D** Polar Husky

Dogs have been bred to specialize in various jobs since their domestication, including pulling sleds to transport goods and people in arctic conditions [1, 2]. Over the centuries, sledding dogs have evolved into distinct populations, some being recognized as purebred breeds like the Siberian Husky and Alaskan Malamute, while others have maintained a less formal status but are commonly known, including the Inuit dog and Alaskan sled dog. Additionally, there are less common sled dog populations, including Polar Huskies, that have had very limited or no study but are identified as unique compared to the more common sled dogs by owners and breeders. Although distinct populations or breeds, sled dogs share a common ancestry and have similar traits selected for breeding [3]. Therefore, it is likely that the various sled dog populations carry many of the same health traits derived from common ancestral origins. Figure 1 shows representative dogs of the three populations of sled dogs assessed in this study, with variation in coat color and length, stature, and ear conformation being particularly noticeable.

### Siberian Husky

Siberian Huskies are known for their aloof behavior and are considered independent, often making them a more challenging breed to train [4]. They are also known for their physical prowess and ability to work in extreme conditions and are considered strong runners over long distances [5]. Due to their status as an American Kennel Club (AKC) recognized breed, Siberian Huskies are subject to strict selection on their appearance and any performance characteristics for kennels that continue to race them, which has led to a closed breeding population [6]. Siberian Huskies are a popular dog breed in the United States and represent the 14^th^ most popular purebred dog in 2020 [7]. Siberian Huskies diverged from other dog breeds early on in domestication and represent, along with Alaskan Malamutes, the ancient origin of many northern dog breeds [8, 9].

### Alaskan sled dogs

Alaskan sled dogs are an admixed breed selected primarily on performance [5]. Initially bred for transportation, the breed transitioned to racing with the advent of mechanical transport in the arctic [2]. As races diverged into different lengths, two distinct subpopulations of Alaskan sled dogs emerged, sprint and distance. Sprint dogs specialize in reaching speeds of 25 to 40 km per hour for distances of less than 50 km. Distance dogs run much longer races such as the Iditarod or Yukon Quest, which span 1,600 km over days or weeks at speeds of 10 to 20 km per hour. In all, Alaskan sled dogs are a genetically distinct population of dogs based upon intense selection for performance despite their mixed breed ancestry and unrestricted breeding program. In addition to the genetic pattern unique to Alaskan sled dogs, both performance subpopulations show genetic ancestry, including Siberian Husky and Alaskan Malamute. While these two arctic breeds are the predominant purebred genetic signatures found in the distance sled dogs, the dogs bred for sprint racing tend to have ancestry that includes Pointers and Salukis as well [5]. Within Alaskan sled dogs, the performance subpopulations show genetic divergence likely reflecting variation in ancestry and admixture as well as prioritization of speed versus endurance [5, 10].

### Polar Husky

Polar Huskies encompass a rare group of dogs currently used for modern arctic exploration. Similar to Alaskan sled dogs, they are not a registered breed and have an unrestricted breeding program. Polar Huskies have not been previously characterized phenotypically or genetically but are commonly expected to be of larger stature and weight (averaging 40 kg). They exhibit various phenotypes but can reach two meters tall standing on hind legs and have a variety of possible colorations [11]. These dogs do not belong to the recognized Greenland dog, nor Inuit dog breeds, yet resemble these ancient breeds used for Arctic exploration and transport in remote or extreme conditions [12].

Embark works as a direct-to-consumer genomic testing service. This study’s goal was to describe the ancestral haplogroups and haplotypes, breed makeup, and health trait carrier status for three sled dog populations sampled for various projects and genotyped using the Embark testing services' information. Insight provided from the Embark panel not only can be used for breed descriptions but also specify genetic variants and health traits that are present in our populations. Understanding the prevalence of diseases can also help identify where the traits may have been introduced based on the ancestry of the carriers. This information can be utilized by both veterinarians and breeders to manage the health of the kennels.

## Results

This project analyzed an opportunistic dataset consisting of 346 Alaskan sled dogs, 13 Polar Huskies, and 89 Siberian Huskies from 61 different kennels genotyped using Embark for other research projects. Of the 346 Alaskan sled dogs, 208 were competitive sprint racing dogs, with 11 additional dogs categorized as sprint due to the mileage they commonly ran but were used for hobby sledding. The remaining Alaskan sled dogs fell into traditional distance or mid-distance categories. The Polar Huskies were all from a single kennel. The 89 AKC registered Siberian Huskies represented kennels selecting dogs for use in show competition, for show and sledding, for competitive distance racing, and as pet dogs. The owner reported breed and racing type of Alaskan sled dog was used to define dog categories within the various analyses.

### Embark breed identification, Mt and Y chromosome haplogroups/types, and coefficient of inbreeding

**Fig. 2:**
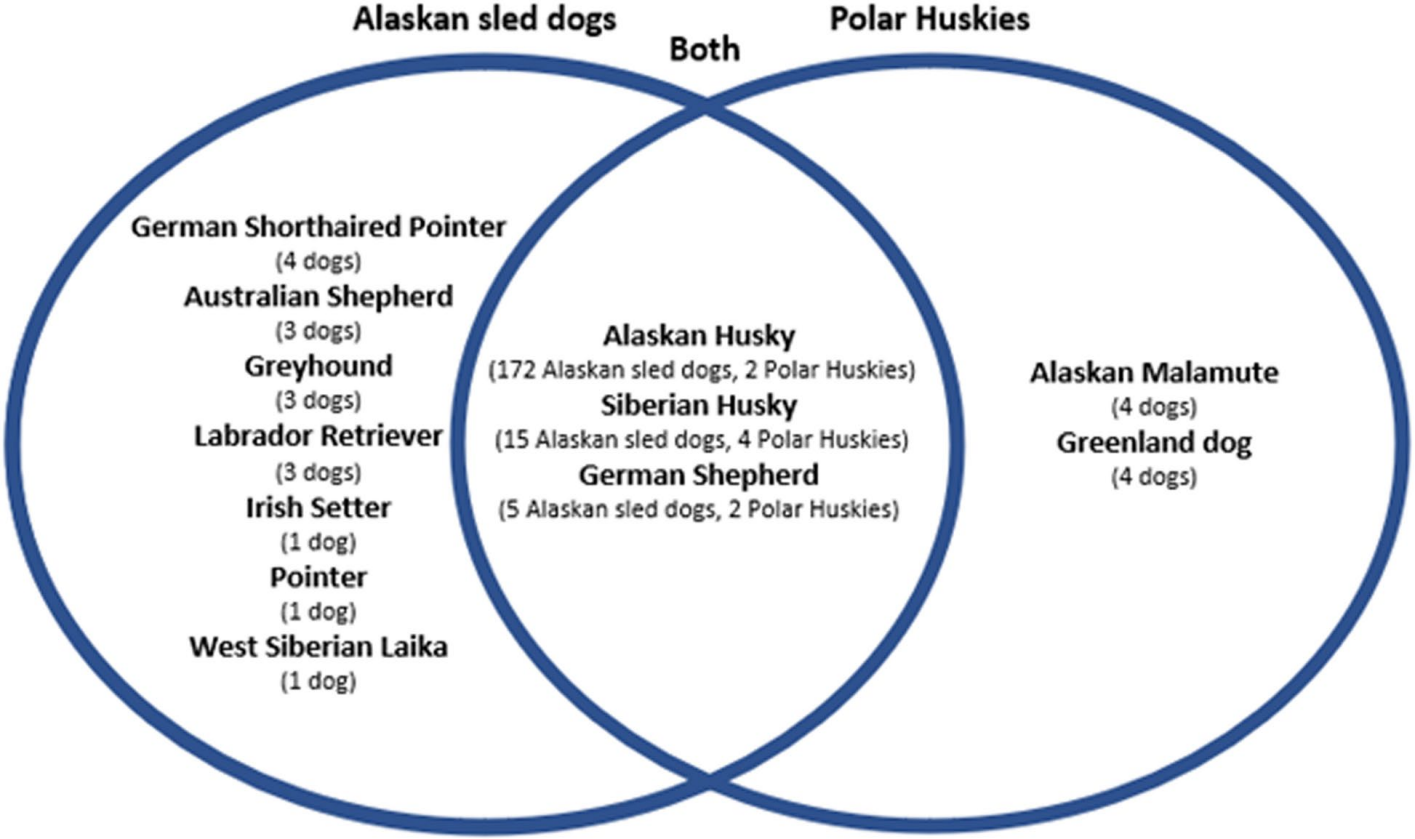
Count of Alaskan sled dogs and Polar Huskies with Ancestry from Different Breeds. A Venn diagram with the number of dogs with ancestry of each breed listed in parenthesis. Individual dogs are counted in multiple breed ancestry populations when they are assigned mixed-breed ancestry

**Fig. 3:**
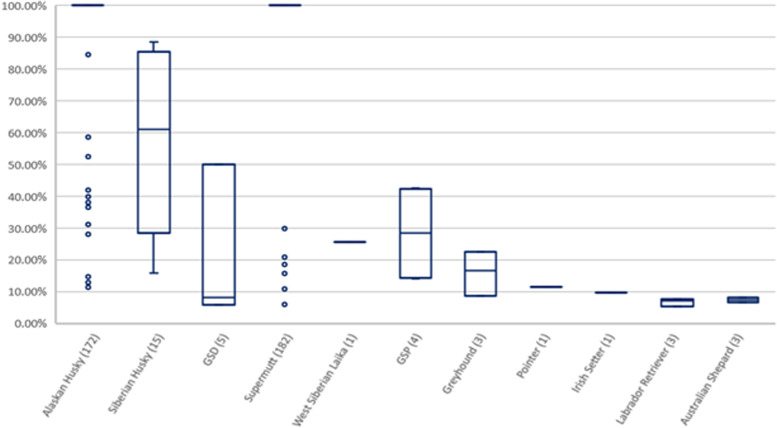
The Average Percentage of Breed-Makeup in Alaskan sled dogs. Each breed boxplot reflects the variation of the specified breed within the Alaskan sled dogs identified as having that breed ancestry. Parentheses following the ancestral breed group denote the number of Alaskan sled dogs having a particular breed ancestry. Individual dogs are counted in multiple breed ancestry populations when they are assigned mixed-breed ancestry. The boxplots display the median, 1^st^ and 3^rd^ quartiles with outliers as circles. *GSD stands for German Shepherd Dog *GSP stands for German Shorthaired Pointer

**Fig. 4:**
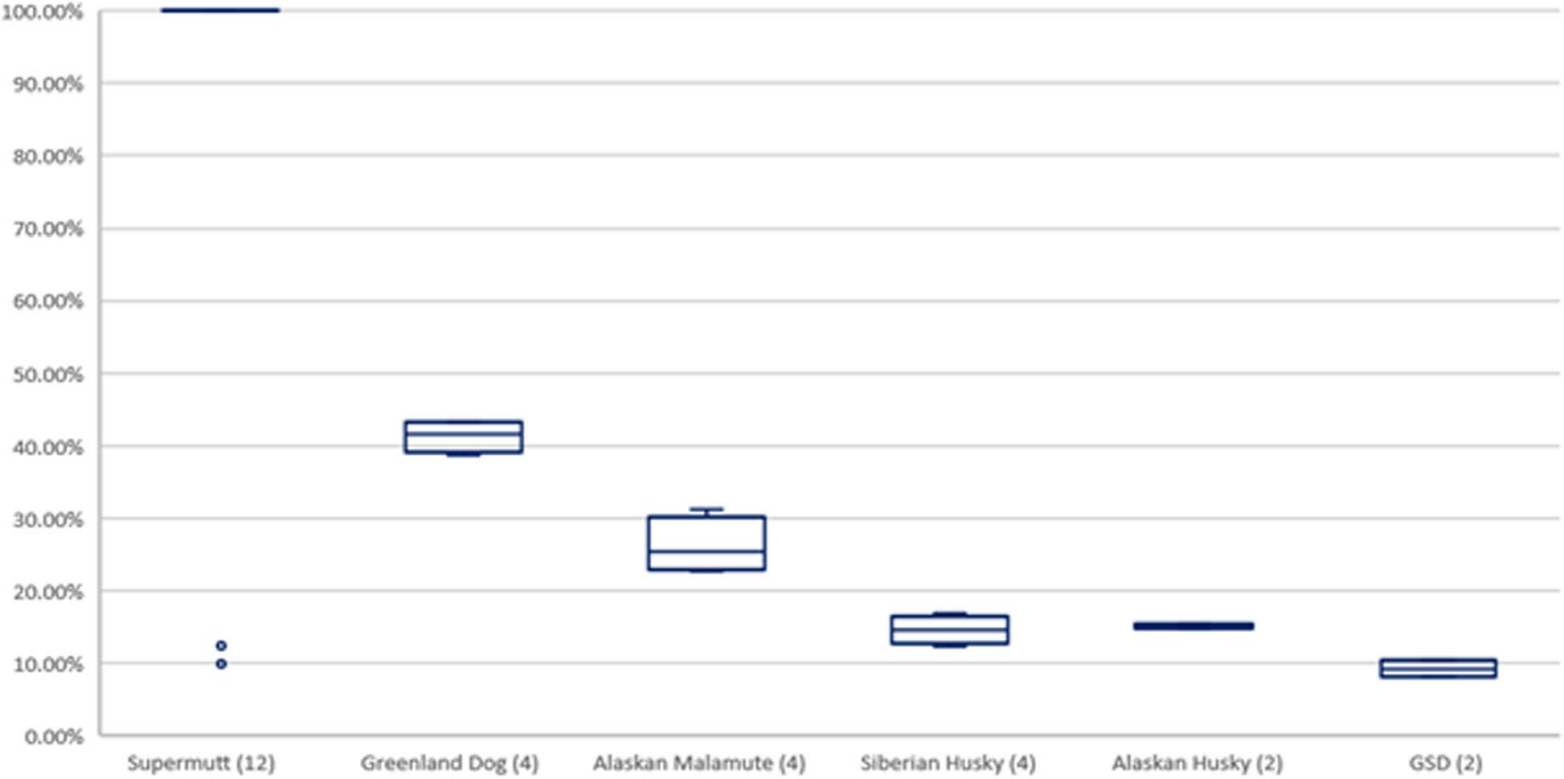
The Average Percentage of Breed-Makeup in Polar Huskies Contributed by Breed. Each breed boxplot reflects the variation of the specified breed within the Polar Huskies identified as having that breed ancestry. The number of Polar Huskies having a particular breed ancestry is denoted in parenthesis behind the ancestral breed group. Individual dogs are counted in multiple breed ancestry populations when they are assigned mixed-breed ancestry. The boxplots display the median, 1^st^ and 3^rd^ quartiles with outliers as circles. *GSD stands for German Shepherd Dog

Breed composition was determined for the admixed populations of Alaskan sled dogs and Polar Huskies. All dogs identified as Siberian Huskies had 100% Siberian Husky ancestry apart from one individual that was 50% Siberian Husky, 50% Alaskan Husky type. Figure 2 shows the number of dogs with a given breed in the Alaskan sled dog and Polar Husky populations. Alaskan sled dogs had 11 breeds represented, and Polar Huskies had six breeds. Trace breeds were also found and were defined as having contributed less than 5% to the overall breed composition. Among the Alaskan sled dogs, trace breeds included: American Eskimo Dog, Alaskan Klee Kai, Belgian Malinois, Belgian Sheepdog, Bergamasco Sheepdog, Border Collie, Collie, Dalmatian, English Setter, English Springer Spaniel, German Wirehaired Pointer, German Longhaired Pointer, Greenland Dog, Irish Red, and White Setter Munsterlander, Pudelpointer, Keeshond, Rottweiler, Saluki, Vizsla, Welsh Sheepdog, Whippet, Wirehaired Pointing Griffon, and Yakutian Laika. Trace breeds for the Polar Huskies were: Alaskan Klee Kai, Bohemian Shepherd, Chinook, and German Shorthaired Pointer. The average percentage of breed-mix contributed by each purebred in Alaskan sled dogs is shown in Fig. 3, and the corresponding data for Polar Huskies is in Fig. 4. For example, of the 172 Alaskan sled dogs with Alaskan Husky Type breed ancestry, the vast majority of these Alaskan sled dogs were considered 100% Alaskan Husky (Figs. 2 and 3). In contrast, 15 Alaskan sled dogs were identified as having Siberian Husky ancestry, but the contribution of this purebred breed to the 15 Alaskan sled dogs varied considerably from 30–85% within individual dogs (Figs. 2 and 3). Dogs from distance racing kennels were far more homogenous than sprint racing dogs in their breed makeup with almost all being 100% Supermutt/Alaskan Husky with most of the breed diversity seen coming from sprint racing dogs. This is consistent with breeding practices and known pedigree information in that sprint racing kennels more commonly crossbreed with purebred breeds to improve speed performance.

Embark also provided the mitochondrial and Y haplogroup and haplotype for the dogs in the dataset. Among Alaskan sled dogs, 8 mitochondrial haplogroups with 24 haplotypes were found. Among the Polar Huskies 3 mitochondrial haplogroups, each with only 1 haplotype associated, were present and 5 mitochondrial haplogroups with 7 haplotypes were found in Siberian Huskies. Six haplogroups and 11 Y haplotypes were found in Alaskan sled dogs and 3 haplogroups and 4 haplotypes were from the Polar Huskies and Siberian Huskies, respectively. These findings are summarized in Supplemental Table 1.

Each animal had a coefficient of inbreeding assessed and this was averaged to provide an insight into the homozygosity present in the three populations. Alaskan sled dogs had the lowest coefficient of inbreeding (COI) with an average of 0.068, median of 0.053, and standard deviation of 0.046. Polar Huskies had an average COI of 0.136, median of 0.078, and standard deviation of 0.114. The Siberian Huskies sampled had an average inbreeding of 0.206, median of 0.197, and standard deviation of 0.088.

### Principal component analysis

**Fig. 5:**
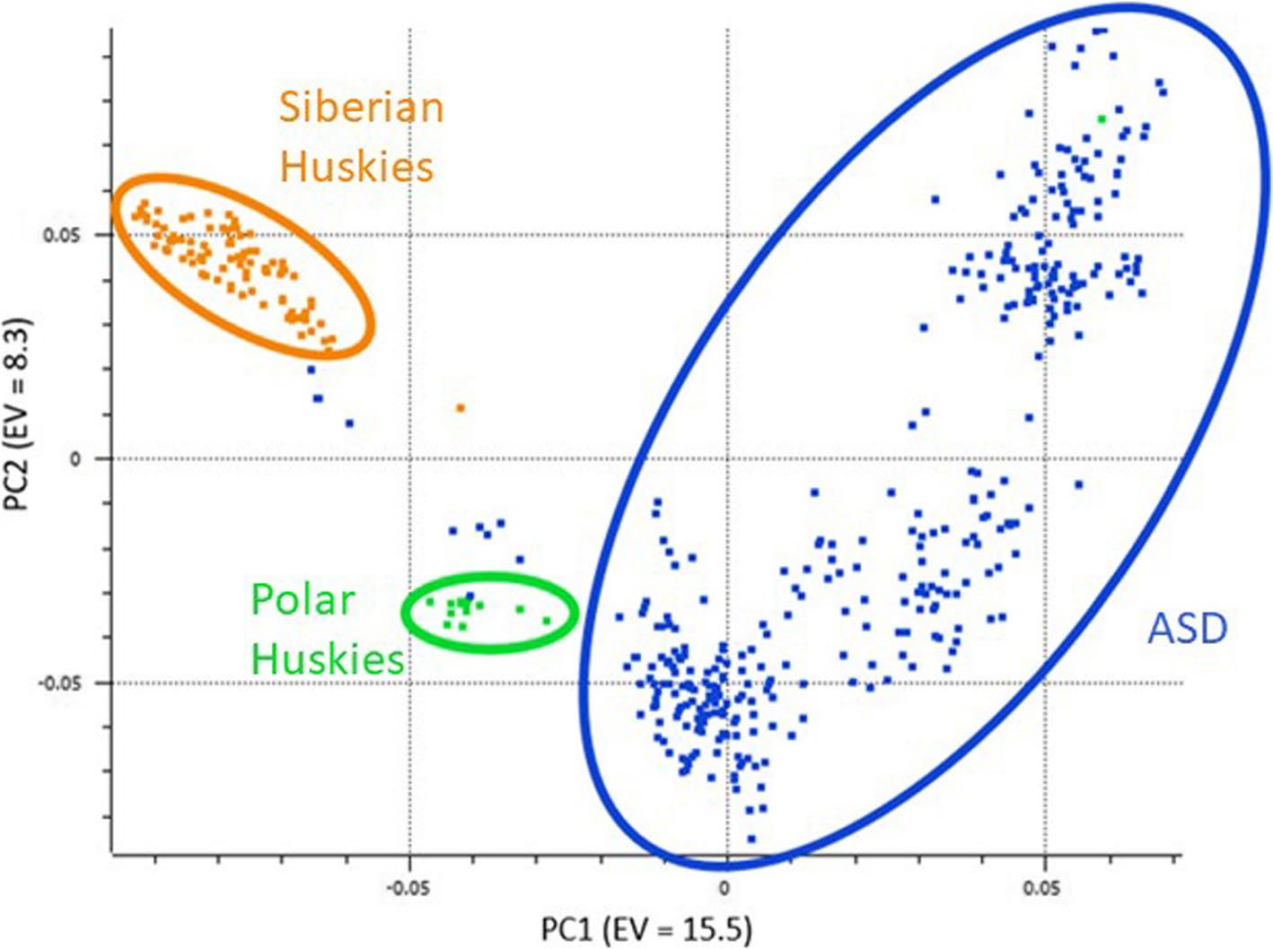
Genomic principal component analysis of the three sled dog populations. Individual dogs are colored based upon the breed they were identified as upon sampling; Siberian Huskies (orange), Polar Huskies (green), and Alaskan sled dogs (blue). PC1 is displayed along the x-axis and PC2 along the y-axis. Eigenvalues are listed in parenthesis. Circles provide a general outline of the distribution of dogs within each population. *ASD stands for Alaskan sled dog

**Fig. 6:**
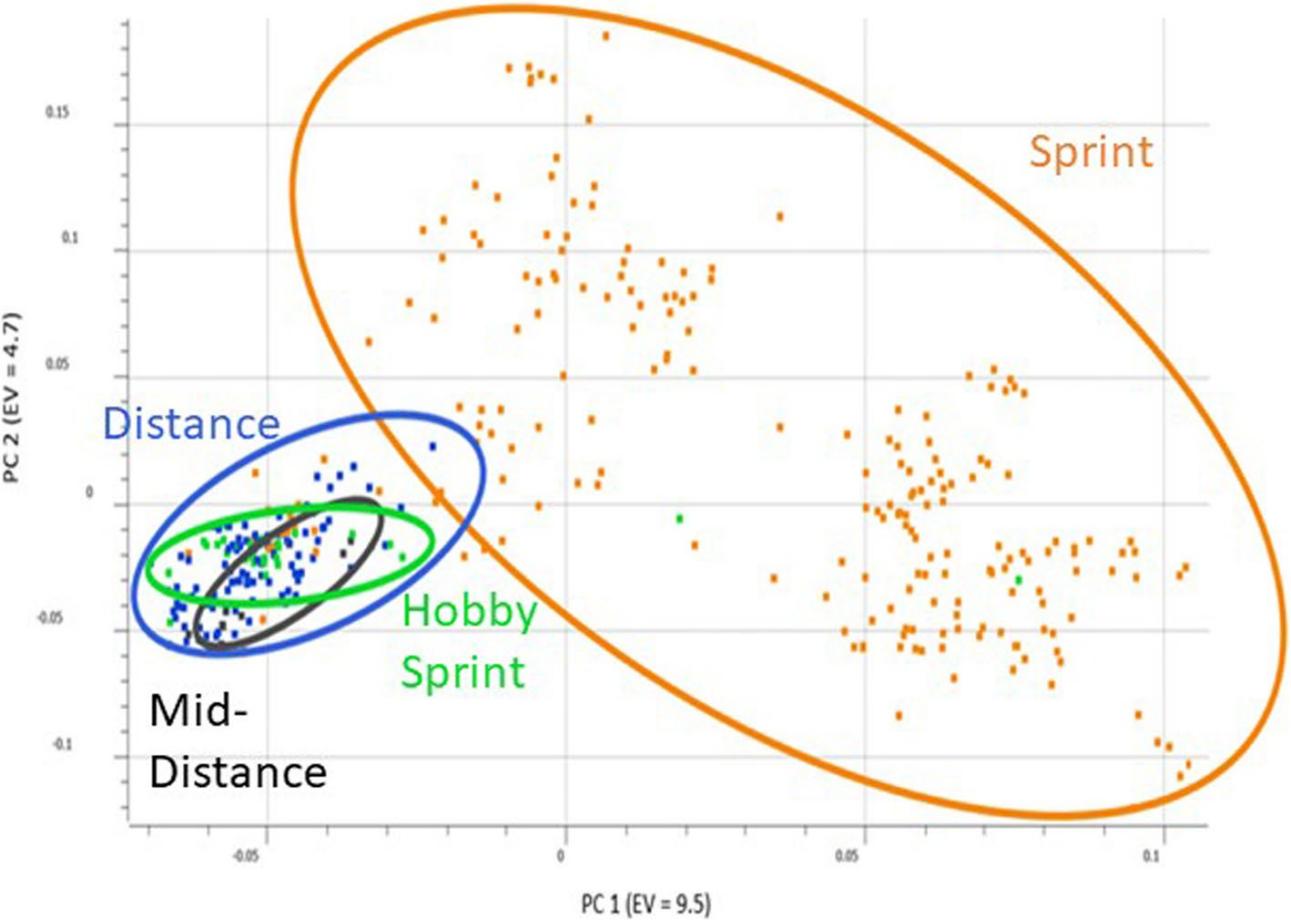
Genomic Principal Component Analysis of the Alaskan sled dog population. Individual dogs are colored based upon the racing type they were identified as upon sampling; sprint (orange), hobby sprint (green), mid-distance (black), distance (blue). Circles provide a general outline of the distribution of dogs within each population. PC1 is displayed along the x-axis and PC2 along the y-axis. Eigenvalues are listed in parenthesis

The population structure of dogs was found using Principal Component Analyses: Figs. 5 and 6 display PC1 along the x-axis and PC2 along the y-axis. Figure 5 represents the total population of dogs, colored by their breed, depicting population structure reflective of sledding breed. Polar Huskies clustered intermediate to Alaskan sled dogs and Siberian Huskies. Overall, Siberian Huskies grouped in a relatively tight cluster, demonstrating more similarity among individuals as expected within a purebred breed. In contrast, the Alaskan sled dogs demonstrated substantial genetic variation with broad clustering likely reflecting population substructure, increased genetic variation due to their open breeding program, and the greater number of individuals in this group. A few Alaskan sled dogs lie outside of the main group of Alaskan sled dogs and closer to Polar or Siberian Huskies. To determine the influence of population size on structure, random subsets of 13 dogs representing each sledding group were similarly analyzed and showed the same general relationship patterns. Figure 6, showing only Alaskan sled dogs, confirms the population substructure within Alaskan sled dogs based on racing type of sprint or distance. Mid-distance dogs clustered within the distance dogs. Interestingly, most of the hobby sprint dogs and some sprint dogs also clustered within the distance group whereas only one distance dog was within the main cluster of sprint dogs.

### Health traits

Along with ancestral results, Embark provides information on more than 190 different health conditions. Of these, 7 were found in the sledding populations tested. These traits were: alanine aminotransferase (ALT) activity, Alaskan husky encephalopathy (AHE), Collie eye anomaly, degenerative myelopathy, dilated cardiomyopathy, factor VII deficiency, and ichthyosis. The most common health condition was ALT activity. Forty-eight Alaskan sled dogs and 14 Siberian Huskies were homozygous for the lower ALT activity mutation. Of the heterozygous dogs, 167 were Alaskan sled dogs, 2 were Polar Huskies, and 52 were Siberian Huskies. Fifty-one of the total 64 kennels had dogs that carried the allele for lower ALT activity. Twenty Alaskan sled dogs were carriers for Alaskan Husky Encephalopathy. All twenty carriers were Alaskan sled dogs with Embark Alaskan Husky ancestry representing seven different kennels. All three breeds had individuals carrying one copy of the Collie eye anomaly mutation: 3 Alaskan sled dogs, 2 Polar Huskies, and 1 Siberian Husky. A single Alaskan sled dog was a carrier of degenerative myelopathy. Five Alaskan sled dogs were heterozygous for dilated cardiomyopathy and were from three kennels of sprint racing teams. Eight Alaskan sled dogs from 5 kennels were carriers for factor VII deficiency. Four Alaskan sled dog kennels had dogs that carried ichthyosis. Of the 15 carriers, 12 came from a single distance racing kennel. For all health traits, population structure was explored by highlighting carriers in the previously conducted genomic PCA [Supplemental Figures 1–7].

## Discussion

Arctic breed sled dogs have similar histories of breed development and selection as working dogs adapted to arctic conditions. Our results exemplify this with similarities in breed identification, including both admixed Alaskan sled dogs and Polar Huskies having Siberian Husky in their breed composition. In addition, all of the health traits, mitochondrial haplogroups and types, and most of the Y chromosome haplogroups and types found in Siberian and Polar Huskies were present in Alaskan sled dogs. However, each sledding population is unique in its own way. Refined selection for artic exploration, sprint and distance competition, or as a recognized purebred breed has generated corresponding genome variation, allowing for dogs within each arctic breed to be generally distinguished from one another.

### Principal component analysis

The genetic differentiation of sledding breeds is evident based on the distinct clustering of Siberian Huskies, Polar Huskies, and Alaskan sled dogs (Fig. 6). The Alaskan sled dogs represent the largest portion of the dataset and demonstrate greater diversity than the other two breeds. Alaskan sled dogs have a less structured breeding program than Siberian Huskies, along with a larger breeding pool than Polar Huskies, and contribute a substantially larger number of dogs within the dataset than the other two sledding groups. The Polar Huskies tended to group between the purebred Siberian Huskies and the Alaskan sled dogs as expected based upon this breeds’ history of ancestry. All of the Polar Huskies in this study originated from one kennel and have an expectedly tight cluster of dogs, indicating their high degree of similarity. Clustering between Alaskan sled dogs and Siberian Huskies matches their admixed breed composition, which contains some breeds similar to those of Alaskan sled dogs and other, unique components, such as the higher frequency of Alaskan Malamute and Greenland Dog. One individual clustered with the sprint racing Alaskan sled dogs and might represent an individual bred from a different stock than other dogs in the kennel. Among Alaskan sled dogs, the sprint racing dogs are more numerous and diverse than the tighter clustering of the distance dogs, which may be due to the much larger number of kennels of sprint racing dogs representing more of the variability present in the population compared to the smaller number of distance racing kennels represented in this dataset. The clustering difference may also reflect more out-crossing in the sprint racing dogs across kennels, with other purebred breeds, or a combination of both. Both hobby sprint racing dogs and mid-distance dogs were more similar to distance than the essentially dissimilar sprint racing dogs. This close clustering may be due to the hobby sprint and mid-distance kennels utilizing traditional distance racing dogs as breeding stock or the practice of moving sprint racing dogs which are unable to compete at a high level to distance teams. Previous research demonstrates similar clustering of distance and sprint racing dogs [10]. Our results match previous reports showing that breed type, followed by working group are important principal components in dogs [13, 14].

### Breed makeup

Alaskan sled dogs had “Supermutt” as the most common ancestral breed listed, which indirectly supports previous findings establishing that Alaskan sled dogs have their own unique genetic breed signature distinguishing them from other purebred breeds. Given that Embark does not include a population analysis of our dataset to determine if the “Supermutt” pattern identified in the Alaskan sled dog is the same across individuals, we cannot confirm if our supposition is correct. The following most common ancestry was Alaskan Husky, which are the markers present in many village dogs in Alaska that are thought to have descended from sled racing dogs [15] and the first time this breed has been denoted in any commercial ancestry panel to our knowledge. Of the remaining breeds, Siberian Husky and Pointer have both been previously found in Alaskan sled dogs [5, 10]. The Labrador Retriever was found in a single litter of dogs that also had ancestry from German Shepherds. The increased breed diversity in sprint racing dogs matches the more experimental breeding of these kennels as they commonly introduce unique breeds in an effort to increase speed. The trace breeds are more difficult to extrapolate direct purebred ancestry from as the ancestry may indicate shared regions of the genome between breeds with common origins as is the case of many sledding breeds which share an origin from Asia [16].

Polar Huskies, similarly, had “Supermutt” as the most common ancestral breed, but Alaskan Malamute, Greenland Dog, and Siberian Husky were also present. Alaskan Husky and German Shepherd were found only in two dogs. These match expectations as the Polar Huskies are significantly larger than both Siberian Huskies and Alaskan sled dogs, so they would be expected to have ancestry from larger breeds such as the Greenland Dog and Alaskan Malamute, which can help provide thick coats and strong frames for their work.

Alaskan sled dogs had the widest variety in both their mitochondrial and Y haplogroups and haplotypes, likely reflecting the much larger dataset assessed and their open breeding program. This matches the PCA data and breed makeup, indicating that Alaskan sled dogs have the most variation. There was considerable overlap in the haplogroups present in the different sledding breeds. All mitochondrial haplogroups found in Siberian Huskies and Polar Huskies were also present in the Alaskan sled dogs. Polar Huskies also share all of their Y haplogroups with Alaskan sled dogs. However, the most common haplogroup among Siberian Huskies (AY) was absent in Alaskan sled dogs and Polar Huskies. This could indicate a unique paternal lineage that has had a substantial impact in the Siberian Huskies sampled.

The COI for each sampled breed matched what would be expected from what is known of the different breeding practices. Siberian Huskies had the highest COI and the strictest breed standards and registration requirements from a closed breeding population [6]. The average COI found was similar to results previously collected using runs of homozygosity to asses inbreeding in Siberian Huskies which showed their relatively low levels compared to other common purebreds [17]. Polar Huskies represent a smaller group of dogs with a more skewed dataset as evidenced by the difference of the mean and median. This could be due to some individuals within the Polar Husky kennel benefitting from the less structured outbreeding possibilities while others are products of traditional linebreeding due to the small number of dogs available. Alaskan sled dogs had the lowest average COI which may be due to having a larger population of dogs than the Polar Huskies, and a less structured breeding program than the Siberian Huskies. The inbreeding results reflect the population structure seen in the PCA in Fig. 6 where the Alaskan sled dogs reflected the largest degree of variation present compared to the more homogenous Polar Huskies and Siberian Huskies.

### Health traits

Along with breed composition, the dogs in the dataset were also tested for over 190 different genetic health conditions. Regardless of breed composition and which breed the condition was initially identified in, all dogs were tested for the entire list of health trait markers. However, due to their status as working dogs, many of the health traits would be presumed to have strong selection against them, particularly if a trait negatively impacted their performance.

### Alanine aminotransferase activity

While serum ALT activity is a measure of hepatic injury as it can be released in the blood following injury to liver cells [18], previous work identified genotypes associated with decreased, yet within normal canine ranges, of serum ALT activity [18]. This variant, which is not associated with any deleterious medical condition, was found in all three of the sledding breeds. Indeed, it was by far the most common health trait variant found in Alaskan sled dogs and Siberian Huskies with 68 and 75% of the populations carrying at least one copy of the allele respectively. This is roughly double the allele frequency (~ 0.2 versus 0.45 frequency) found in Siberian Huskies as compared to the original research paper identifying the variant. ALT activity was the only condition found in which dogs had heterozygous and both homozygous genotypes present. This may be due to the other mutations being deleterious, which would prevent the dogs from maturing or remaining in the kennels of active sled dogs which were sampled.

### Alaskan husky encephalopathy

AHE, first characterized in the late twentieth century in sled dogs, is a degenerative brain condition that leaves dogs with extreme neurologic dysfunction [19, 20]. The causative mutation, a 4 base pair insertion located on chromosome 25 within the thiamine transporter 2 (*SLC19A3*) gene, controls thiamine transportation in the central nervous system [21]. Ten of the twenty dogs were from a single kennel of hobby sprint racing dogs and all found to carry the variant were Alaskan sled dogs with Alaskan Husky ancestry.

### Collie eye anomaly

Collie eye anomaly is a genetic disorder that causes blood vessels in the eye to be underdeveloped which can lead to detached retinas and blindness [22]. There is a candidate gene, *NHEJ1*, located on chromosome 37 [22]. The condition has previously been identified in Siberian Huskies based on phenotypic presentation [23, 24]. However, in a study of mixed and purebred dogs assessing the frequency of known variants associated to genetic diseases, none of the 111 Siberian Huskies carried the Collie eye anomaly variant, unlike the one carrier in the present study [25]. Two of the Alaskan sled dogs which were carriers originated from a distance racing kennel and one was from a sprint racing kennel. Both Polar Huskies were from the same kennel.

### Degenerative myelopathy

Degenerative myelopathy leads to progressive loss of neurologic function with ataxia and muscle atrophy in the hind limbs [26]. A missense mutation in the *SOD1* gene has been found responsible [27–29]. The condition has previously been described in Siberian Huskies along with over 120 other breeds of dog [25, 26]. Among the dogs in this dataset, only one was found to be a carrier for degenerative myelopathy – a sprint racing Alaskan Sled dog who was a full-bred Alaskan Husky as characterized by Embark.

### Dilated cardiomyopathy

Dogs with dilated cardiomyopathy have enlarged heart chambers and systolic dysfunction [30]. The trait has a dominant inheritance and was initially identified with a variant in the *PDK4* gene in Doberman Pinschers [31]. All the carriers were sprint racing Alaskan sled dogs with three of the five being from the same kennel with mitochondrial haplogroups and haplotypes that suggest a common ancestor may have introduced the gene into that kennel. This matches the PCA results (Supplemental Figure 5) that demonstrate the clustering of carriers among the total population of sledding dogs.

### Factor VII deficiency

Factor VII deficiency is a condition that can lead to increased bleeding due to a variant of the *cFVII* gene, initially found in beagles [32]. The trait has also been identified in Alaskan Klee Kai and German Shorthaired Pointers among many other breeds [25, 33]. Carriers were found among the Alaskan sled dogs; however, none were found to have ancestry from one of the identified breeds as all were full Supermutt or Alaskan Husky.

### Ichthyosis

The ichthyosis mutation was initially identified in Golden Retrievers caused by an indel mutation in the *PNPLA1* gene, although Embark also tests for variants associated with the *NIPAL4* gene from American Bulldogs, the *SLC27A4* gene initially found in Great Danes, and the *FAM83H* gene in Cavalier King Charles Spaniels [34–37]. The disease is characterized by hyperkeratosis and thickening of metapodial and digital pads [38]. In our population, 12 of the carriers were from a single kennel of distance racing sled dogs. Although this trait is considered recessive in other breeds, after the samples were collected the owner of the kennel was asked to create a list of dogs afflicted with "harness rub" which was found to coincide exactly with the dogs that had one copy of the ichthyosis allele. Harness rub is a term used when dogs tend to have the parts of their fur rub off and may appear red or dry where the harness rests on the dogs while running. None of the dogs demonstrated any obvious skin or pad problems according to the owner. This may indicate a potential heterozygotic phenotype which is more susceptible to skin abrasions but requires further study. By highlighting dogs that are carriers in the total PCA in Supplemental Figure 7, the clustering of carriers can be seen, which may indicate a more recent introduction into a small group of dogs as it has not been spread widely within the population.

## Conclusion

Sled dogs are not only a unique group based on evolutionary history but also due to their continued relevance as working dogs. The varied breed makeup of the Polar Huskies and Alaskan sled dogs helps demonstrate the unique genetic histories that have led to the introduction of dogs with different ancestry into their semi-open breeding pools, unlike the closed population among Siberian Huskies. Despite their differences, these breeds act as unique case studies in the different genetic makeup, which can arise due to different breeding strategies. By placing different weights on selection criteria such as uniformity, performance, and size, these dogs have been bred to excel in a variety of different conditions.

An important consideration for this study is the limited size of the dataset as the dogs were sampled over various projects. The Alaskan sled dogs and Polar Huskies sampled, were from kennels which continued to use the dogs as working sled dogs for race or transportation. The Siberian Husky kennels included a wider range of dogs used for show, racing, and as pets. Additionally, health trait assessment was limited to publications of causative SNPs or those in linkage or within haplotypes of causative mutations. While Embark offers the most comprehensive genetic screening of health traits in dogs, there are obviously many unknown and possibly breeds specific variants yet to be discovered. To conduct a more detailed description of the breeds, a larger dataset including other arctic breeds with different working backgrounds can provide new insights into the health and genetic makeup of this family of dogs.

## Materials and methods

### Embark dataset

The aim of this project was to identify and describe the breed composition, mitochondrial and Y chromosome haplogroups and types, and health traits found in three arctic sledding breeds using the commercially available Embark DNA kit. This project used an opportunistic dataset compiling dogs from the three sled dog populations genotyped using Embark for other research projects. This included 346 Alaskan sled dogs, 13 Polar Huskies, and 89 Siberian Huskies from 61 different kennels. Of the 346 Alaskan sled dogs, 208 were competitive sprint racing dogs, with 11 additional dogs categorized as sprint due to the mileage they commonly ran but were used for hobby sledding. The remaining Alaskan sled dogs fell into traditional distance or mid-distance categories. The Polar Huskies were all from a single kennel. The 89 AKC registered Siberian Huskies represented kennels selecting dogs for use in show competition, for show and sledding, for competitive distance racing, and as pet dogs. Following Cornell University’s Institutional Animal Care and Use Committee approval, blood samples were collected for genotyping via either the jugular or cephalic veins, and EDTA was used to prevent clotting. The DNA samples were extracted using standard Qiagen DNA Extraction Protocol®. Each sample's quality and quantity were checked using an Epoch microplate spectrophotometer. The samples were genotyped on the custom Canine HD Illumina BeadChip through Embark, containing 228 k SNPs which is commercially available for research or industry purposes.

### Principal component analysis

A genomic principal component analysis (PCA) was conducted on the total dataset to identify population structure. The SNP data were uploaded to the SNP & Variation Suite v8.8.3 (Golden Helix, Inc., Bozeman, MT, www.goldenhelix.com) to assess genotype quality and conduct the PCA analysis. SNPs were removed based on a call rate of < 0.85, if the number of alleles was > 2, a minor allele frequency of < 0.03, and a Hardy–Weinberg P < 1 × 10^–5^. Samples were removed if they had a call rate less than 0.90. After all quality control filtering was applied, 448 dogs and 144,679 SNPs remained for analysis.

Principal components reflecting genomic variation within the dataset were identified. Eigenvalues described the degree of variation each principal component captured. The PCA utilized an additive genetic model in the analysis. Principal components were plotted for the total dataset and random subsets of the breeds to explore population structure related to breed and health trait carrier status. Principal component analysis was also conducted on the Alaskan sled dogs alone, as they comprise the largest subset of animals within the dataset and have a known history of population substructure related to racing style [10].

### Embark breed composition

Embark utilizes results from registered purebred dogs to identify unique markers that can assign breed composition to dogs added to their database. There are currently over 350 breeds tested by the service [39]. Percentages are assigned to individuals based on the proportion of ancestry that a breed contributed. The “Supermutt” breed denotes regions not identified as belonging to a single ancestral breed. Any breed markers identified as contributing less than 5% of the ancestry are listed as ‘Trace Breeds’ [40]. Along with purebred dogs, Embark also has data from village dogs around the world who display a unique pattern of genetic markers. Of note, there is a population of dogs from Alaskan villages, denoted as Alaskan Husky-type dogs.

### Embark mitochondrial and Y chromosome haplogroups and haplotypes

Haplotypes can help group dogs of similar ancestry by identifying common series of inherited markers. Haplogroups are composed of series of related haplotypes [41]. Embark provides both the Y chromosome haplogroup and haplotype, which is indicative of paternal lineage, and the mitochondrial haplogroup and haplotype indicative of the maternal lineage [42].

### Embark coefficient of inbreeding

The genetic coefficient of inbreeding can provide an insight into the relatedness of ancestors of an individual dog. Embark analyzes runs of homozygous markers of at least 1 centimorgan in length to determine the COI [43]. This can provide a more accurate measure of inbreeding than either pedigree or other marker based techniques [44, 45].

### Embark health traits

The Embark panel creates a health assessment for dogs based upon the alleles they possess by characterizing single-nucleotide polymorphisms that are either directly responsible for or strongly linked with health traits. Dogs are denoted as ‘carrier,’ having one copy of an allele, or ‘clear,’ having no copies of a target allele. Embark categorizes health traits as: clinical, blood, hormones, immune, eyes, kidney and bladder, multisystem, other systems, brain and spinal cord, heart, muscular, metabolic, gastrointestinal, neuromuscular, skin & connective tissues, and skeletal [46]. Original research publications of each disease variant are referenced on the Embark website within the individual disease description pages.

Embark provides the breed composition, Y and MT haplogroups, types, inbreeding, and carrier status for genetic health traits as part of the genetic testing service. It was this commercially provided information, which was our focus in describing the three arctic sled dog populations. This information was analyzed using R and supported by genomic principal component analysis to assess population structure.

## Supplementary Information

**Additional file 1: Supplemental Table 1.** The distribution of dogs within Arctic sledding populations belonging to various haplogroups and haplotypes. The haplogroups identified by Embark for each sledding population are denoted under the Haplogroup column. Population haplotypes within each haplogroup are adjacent to the named haplogroup.

**Additional file 2: Supplemental Figure 1.** Genomic Principal Component Analysis of All Sledding Populations by Lower than Normal ALT Carrier Status. Individual dogs are colored based upon their carrier status for lower than normal ALT with homozygous carriers displayed in green, heterozygous dogs in blue, homozygous clear dogs in orange and dogs without data in black. PC1 is displayed along the x-axis and PC2 along the y-axis. Eigenvalues are listed in parenthesis. Circles and corresponding text reference the general distribution of individuals within the three populations. *ASD stands for Alaskan sled dog.

**Additional file 3: Supplemental Figure 2.** Genomic Principal Component Analysis of All Sledding Populations by Alaskan Husky Encephalopathy Carrier Status. Individual dogs are colored based upon their carrier status for Alaskan Husky encephalopathy with carriers displayed in blue, clear dogs in green, and dogs without data in orange. PC1 is displayed along the x-axis and PC2 along the y-axis. Eigenvalues are listed in parenthesis. Circles and corresponding text reference the general distribution of individuals within the three populations. *ASD stands for Alaskan sled dog.

**Additional file 4: Supplemental Figure 3.** Genomic Principal Component Analysis of All Sledding Populations by Collie Eye Anomaly Carrier Status. Individual dogs are colored based upon their carrier status for Collie eye anomaly with carriers displayed in blue, clear dogs in green, and dogs without data in orange. PC1 is displayed along the x-axis and PC2 along the y-axis. Eigenvalues are listed in parenthesis. Circles and corresponding text reference the general distribution of individuals within the three populations. *ASD stands for Alaskan sled dog.

**Additional file 5: Supplemental Figure 4.** Genomic Principal Component Analysis of All Sledding Populations by Degenerative Myelopathy. Individual dogs are colored based upon their carrier status for degenerative myelopathy with carriers displayed in blue (within the larger blue circle for easier identification), clear dogs in green, and dogs without data in orange. PC1 is displayed along the x-axis and PC2 along the y-axis. Eigenvalues are listed in parenthesis. Circles and corresponding text reference the general distribution of individuals within the three populations. *ASD stands for Alaskan sled dog.

**Additional file 6: Supplemental Figure 5.** Genomic Principal Component Analysis of All Sledding Populations by Dilated Cardiomyopathy Carrier Status. Individual dogs are colored based upon their carrier status for dilated cardiomyopathy with carriers displayed in blue (within the larger blue circle for easier identification), clear dogs in green, and dogs without data in orange. PC1 is displayed along the x-axis and PC2 along the y-axis. Eigenvalues are listed in parenthesis. Circles and corresponding text reference the general distribution of individuals within the three populations. *ASD stands for Alaskan sled dog.

**Additional file 7: Supplemental Figure 6.** Genomic Principal Component Analysis of All Sledding Populations by Factor VII Deficiency. Individual dogs are colored based upon their carrier status for factor VII deficiency with carriers displayed in blue, clear dogs in green, and dogs without data in orange. PC1 is displayed along the x-axis and PC2 along the y-axis. Eigenvalues are listed in parenthesis. Circles and corresponding text reference the general distribution of individuals within the three populations. *ASD stands for Alaskan sled dog.

**Additional file 8: Supplemental Figure 7.** Genomic Principal Component Analysis of All Sledding Populations by Ichthyosis Carrier Status. Individual dogs are colored based upon their carrier status for ichthyosis with carriers displayed in blue (within the larger blue circle for easier identification) and dogs without data in orange. PC1 is displayed along the x-axis and PC2 along the y-axis. Eigenvalues are listed in parenthesis. Circles and corresponding text reference the general distribution of individuals within the three populations. *ASD stands for Alaskan sled dog.

## Data Availability

The datasets analyzed during the current study are not publicly available due to individual owner consent restrictions and accessibility of research data within the Embark system but are available from the corresponding author on reasonable request and owner consent.

## Abbreviations

AKC: American Kennel Club
ALT: Alanine Aminotransferase
AHE: Alaskan Husky Encephalopathy
COI: Coefficient of Inbreeding
MT: Mitochondrial
SNP: Single Nucleotide Polymorphism
