# Supplementary figures and images for: Description of breed ancestry and genetic health traits in arctic sled dog breeds
Source: Canine Med Genet. 2021 Sep 20;8:8. doi: 10.1186/s40575-021-00108-z (PMC8454093; doi:10.1186/s40575-021-00108-z)

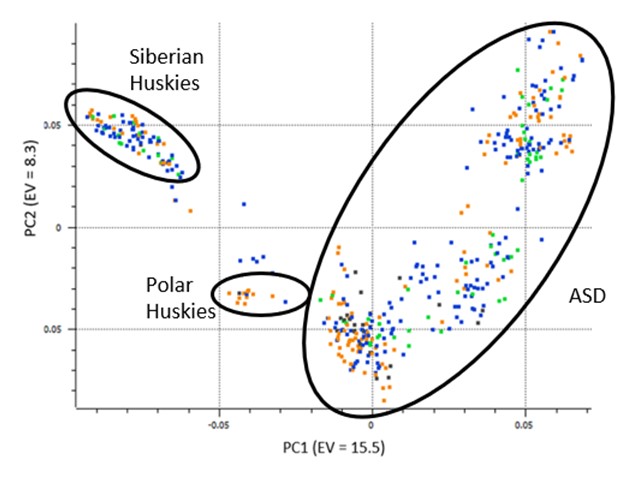

Supplement: Supplementary file 2 — Additional file 2: Supplemental Figure 1. Genomic Principal Component Analysis of All Sledding Populations by Lower than Normal ALT Carrier Status. Individual dogs are colored based upon their carrier status for lower than normal ALT with homozygous carriers displayed in green, heterozygous dogs in blue, homozygous clear dogs in orange and dogs without data in black. PC1 is displayed along the x-axis and PC2 along the y-axis. Eigenvalues are listed in parenthesis. Circles and corresponding text reference the general distribution of individuals within the three populations. *ASD stands for Alaskan sled dog. [file 40575_2021_108_MOESM2_ESM.jpg]

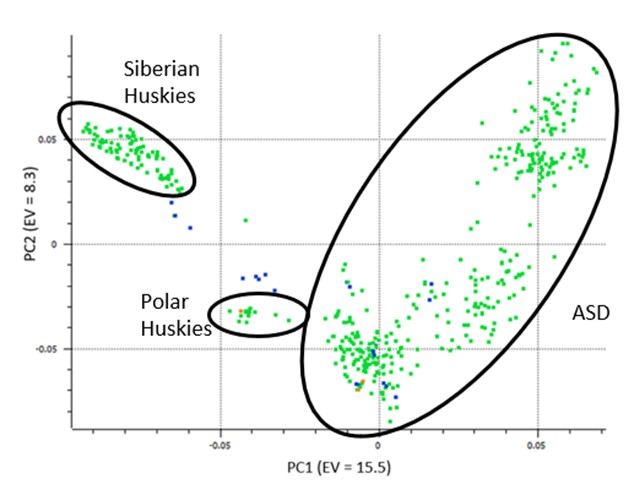

Supplement: Supplementary file 3 — Additional file 3: Supplemental Figure 2. Genomic Principal Component Analysis of All Sledding Populations by Alaskan Husky Encephalopathy Carrier Status. Individual dogs are colored based upon their carrier status for Alaskan Husky encephalopathy with carriers displayed in blue, clear dogs in green, and dogs without data in orange. PC1 is displayed along the x-axis and PC2 along the y-axis. Eigenvalues are listed in parenthesis. Circles and corresponding text reference the general distribution of individuals within the three populations. *ASD stands for Alaskan sled dog. [file 40575_2021_108_MOESM3_ESM.jpg]

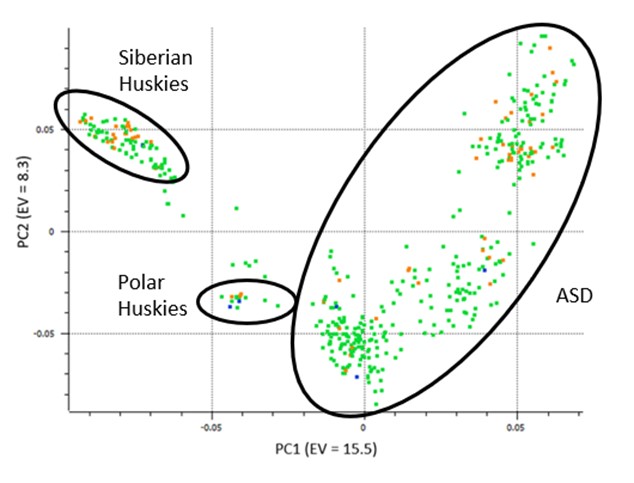

Supplement: Supplementary file 4 — Additional file 4: Supplemental Figure 3. Genomic Principal Component Analysis of All Sledding Populations by Collie Eye Anomaly Carrier Status. Individual dogs are colored based upon their carrier status for Collie eye anomaly with carriers displayed in blue, clear dogs in green, and dogs without data in orange. PC1 is displayed along the x-axis and PC2 along the y-axis. Eigenvalues are listed in parenthesis. Circles and corresponding text reference the general distribution of individuals within the three populations. *ASD stands for Alaskan sled dog. [file 40575_2021_108_MOESM4_ESM.jpg]

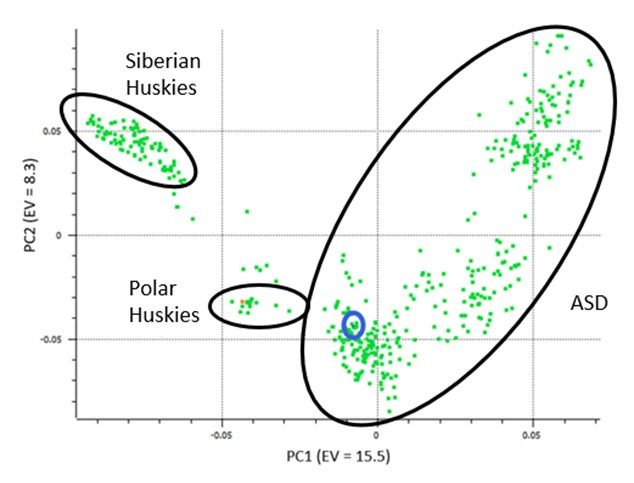

Supplement: Supplementary file 5 — Additional file 5: Supplemental Figure 4. Genomic Principal Component Analysis of All Sledding Populations by Degenerative Myelopathy. Individual dogs are colored based upon their carrier status for degenerative myelopathy with carriers displayed in blue (within the larger blue circle for easier identification), clear dogs in green, and dogs without data in orange. PC1 is displayed along the x-axis and PC2 along the y-axis. Eigenvalues are listed in parenthesis. Circles and corresponding text reference the general distribution of individuals within the three populations. *ASD stands for Alaskan sled dog. [file 40575_2021_108_MOESM5_ESM.jpg]

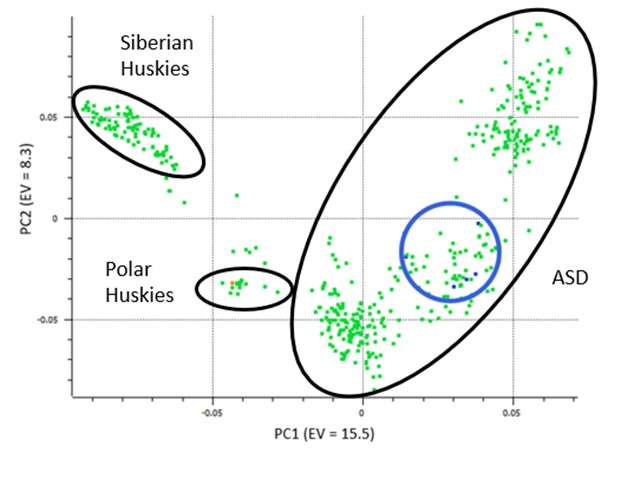

Supplement: Supplementary file 6 — Additional file 6: Supplemental Figure 5. Genomic Principal Component Analysis of All Sledding Populations by Dilated Cardiomyopathy Carrier Status. Individual dogs are colored based upon their carrier status for dilated cardiomyopathy with carriers displayed in blue (within the larger blue circle for easier identification), clear dogs in green, and dogs without data in orange. PC1 is displayed along the x-axis and PC2 along the y-axis. Eigenvalues are listed in parenthesis. Circles and corresponding text reference the general distribution of individuals within the three populations. *ASD stands for Alaskan sled dog. [file 40575_2021_108_MOESM6_ESM.jpg]

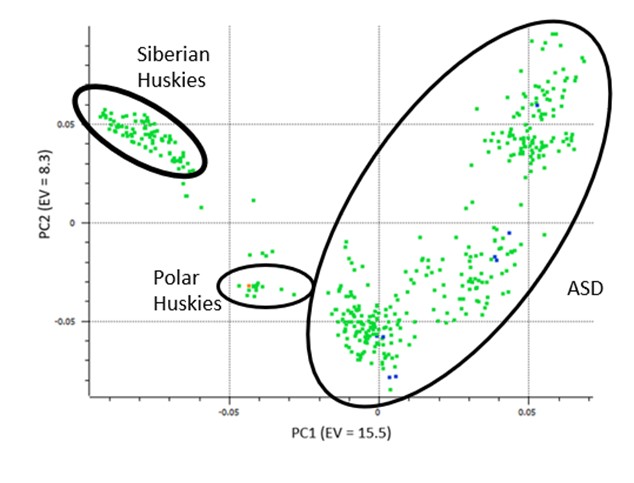

Supplement: Supplementary file 7 — Additional file 7: Supplemental Figure 6. Genomic Principal Component Analysis of All Sledding Populations by Factor VII Deficiency. Individual dogs are colored based upon their carrier status for factor VII deficiency with carriers displayed in blue, clear dogs in green, and dogs without data in orange. PC1 is displayed along the x-axis and PC2 along the y-axis. Eigenvalues are listed in parenthesis. Circles and corresponding text reference the general distribution of individuals within the three populations. *ASD stands for Alaskan sled dog. [file 40575_2021_108_MOESM7_ESM.jpg]

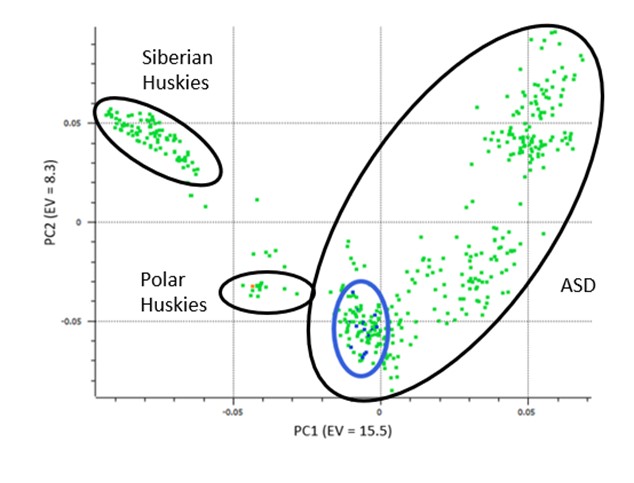

Supplement: Supplementary file 8 — Additional file 8: Supplemental Figure 7. Genomic Principal Component Analysis of All Sledding Populations by Ichthyosis Carrier Status. Individual dogs are colored based upon their carrier status for ichthyosis with carriers displayed in blue (within the larger blue circle for easier identification) and dogs without data in orange. PC1 is displayed along the x-axis and PC2 along the y-axis. Eigenvalues are listed in parenthesis. Circles and corresponding text reference the general distribution of individuals within the three populations. *ASD stands for Alaskan sled dog. [file 40575_2021_108_MOESM8_ESM.jpg]
